# A technique for penetrating the bile duct wall using a guidewire during endoscopic ultrasound-guided hepaticogastrostomy

**DOI:** 10.1055/a-2537-0534

**Published:** 2025-02-26

**Authors:** Takeshi Ogura, Kimi Bessho, Takafumi Kanadani, Nobuhiro Hattori, Hiroki Nishikawa

**Affiliations:** 1 Endoscopy Center, Osaka Medical and Pharmaceutical University Hospital, Takatsuki, Japan; 2 2nd Department of Internal Medicine, Osaka Medical and Pharmaceutical University, Takatsuki, Japan

Endoscopic ultrasound-guided hepaticogastrostomy (EUS-HGS) involves several technical steps, such as intrahepatic bile duct puncture, guidewire insertion into the biliary tract, tract dilation, and stent deployment 1 2 3 . Intrahepatic bile duct puncture is usually performed using a 19-G needle because use of a 22-G needle also requires the use of a 0.018-inch guidewire. However, when using a 19-G needle, puncturing the bile duct wall may be challenging if it is extremely thick. In such cases, the Seldinger technique combined with the door knocking method is sometimes used, but there is a risk of vessel injury. To overcome this, a technique for penetrating the bile duct wall using a guidewire is described.

An 88-year-old patient underwent EUS-guided antegrade metal stent deployment for distal biliary obstruction because of surgically altered anatomy due to cancer of the head of the pancreas, and EUS-HGS was performed using a plastic stent. However, 6 months later, recurrent biliary obstruction was observed due to antegrade metal stent obstruction and EUS-HGS stent dislocation. Therefore, EUS-HGS was attempted.

On EUS imaging, a thickened bile duct wall was observed because an EUS-HGS stent had been previously deployed ( Fig. 1 **a** ), and intrahepatic bile duct puncture with standard puncture and the Seldinger technique using a 19-G needle failed. Therefore, a 19-G needle was advanced to the bile duct wall ( Fig. 1 **b** ), and the bile duct wall was successfully penetrated using a 0.025-inch guidewire ( Fig. 1 **c** ). To prevent guidewire insertion into the hepatic parenchyma or vessels, the guidewire should be continuously identified on EUS imaging. After guidewire deployment, a double-lumen dilator was inserted into the biliary tract ( Fig. 2 **a** ). An additional guidewire was inserted through this dilator to perform a double-guidewire technique, and the stent delivery system was inserted. Finally, metal stent deployment from the intrahepatic bile duct to the stomach was successfully performed without any adverse events ( Fig. 2 **b** , Video 1 ).

**Fig. 1:**
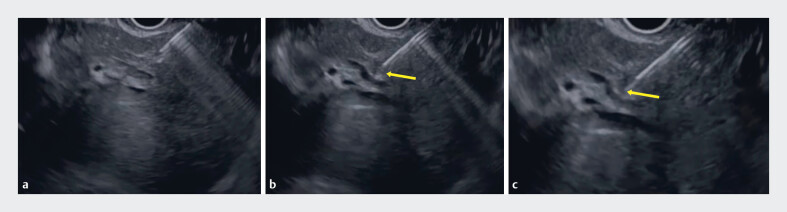
Endoscopic ultrasound images. **a** A thickened bile duct wall was observed. **b** A 19-G needle (arrow) was advanced to the bile duct wall. **c** The bile duct wall was successfully penetrated using a 0.025-inch guidewire (arrow).

**Fig. 2:**
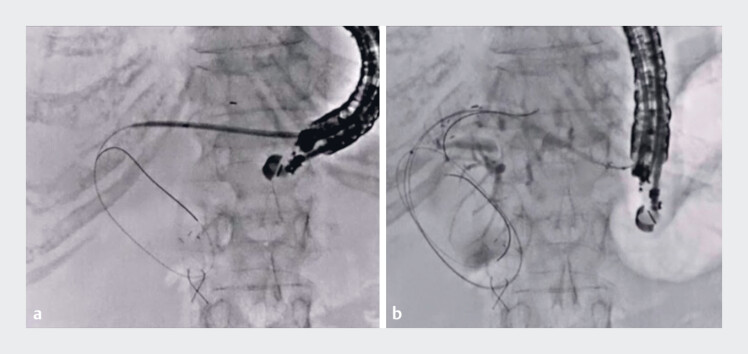
Fluoroscopic images. **a** A double-lumen dilator was inserted into the biliary tract. **b** Metal stent deployment from the intrahepatic bile duct to the stomach was successfully performed.

A technique for penetrating the bile duct wall using a guidewire during endoscopic ultrasound-guided hepaticogastrostomy.Video 1

In conclusion, the technique described may facilitate guidewire insertion for cases in which bile duct puncture is challenging.

Endoscopy_UCTN_Code_TTT_1AS_2AH

## Notes

Endoscopy E-Videos https://eref.thieme.de/e-videos
*E-Videos* is an open access online section of the journal *Endoscopy* , reporting on interesting cases and new techniques in gastroenterological endoscopy. All papers include a high-quality video and are published with a Creative Commons CC-BY license. Endoscopy E-Videos qualify for HINARI discounts and waivers and eligibility is automatically checked during the submission process. We grant 100% waivers to articles whose corresponding authors are based in Group A countries and 50% waivers to those who are based in Group B countries as classified by Research4Life (see: https://www.research4life.org/access/eligibility/ ). This section has its own submission website at https://mc.manuscriptcentral.com/e-videos .
